# A novel deep learning approach for privacy-preserving encoded EEG-based brain-computer interfaces with clinical LLM applications

**DOI:** 10.1007/s13755-026-00470-x

**Published:** 2026-07-08

**Authors:** Taslima Khanam, Siuly Siuly, Kate Wang, Hua Wang

**Affiliations:** 1 Institute for Sustainable Industries & Liveable Cities, Victoria University, Melbourne, VIC Australia; 2 Royal Melbourne Institute of Technology (RMIT), Melbourne, VIC Australia

**Keywords:** Electroencephalogram, Brain-computer interfaces, Deep denoising structure-preserving neural encoding network, Neural network, Large language models

## Abstract

**Purpose:**

The rise of large language models (LLMs) such as GPT-4 and DeepSeek has transformed healthcare information processing by enabling natural language-based clinical reasoning. However, the integration of LLMs with privacy-sensitive biomedical signals, particularly electroencephalogram (EEG) data used in brain-computer interface (BCI) systems, remains underexplored. EEG signals, especially during motor imagery (MI) tasks, are critical for assistive neurotechnologies but pose significant privacy risks due to their capacity to reveal cognitive and medical information. Traditional encryption techniques often distort signal structure or require decryption with additional noise, compromising classification performance and real-time usability.

**Methods:**

To address this gap, we propose a deep denoising structure-preserving neural encoding network (DSNet) that enables accurate classification of privacy-preserving encoded EEG representations without requiring decryption. EEG features were extracted using common spatial pattern (CSP) and transformed into privacy-preserving encoded representations while preserving their statistical structure. Here, encoding refers to a non-reversible neural transformation designed for privacy preservation rather than a formal cryptographic guarantee. Two deep learning architectures, a feedforward neural network (NN) and a recurrent neural network (RNN), were evaluated for classification in the encoded feature space. Furthermore, we integrated an LLM (GPT-4) to generate clinical-style summaries based on model outputs, enhancing interpretability for clinician review and potential clinical support use.

**Results and conclusion:**

Using publicly available datasets, DSNet-NN achieved over 87% accuracy for every subject, outperforming both the RNN variant and baseline models. It also demonstrated resilience to simulated privacy attacks. LLM-generated reports provided clinician-friendly interpretations of MI predictions, supporting potential real-world applicability. This study introduces an AI framework that bridges privacy-preserving EEG decoding with LLM-based clinical reasoning, offering a practical solution for privacy-preserving neurorehabilitation and digital health systems.

## Introduction

Recent advances in artificial intelligence (AI), particularly large language models (LLMs) such as ChatGPT and DeepSeek-R1, have significantly transformed healthcare information processing. These models now enable real-time interpretation of clinical narratives, structured health data, and even multimodal biomedical signals. Their ability to generate natural language explanations, annotate clinical outcomes, and support decision making workflows has opened new opportunities for integrating LLMs into clinical support systems. However, most existing research focuses on text and image-based applications, and the extension of LLMs to physiological signal interpretation particularly electroencephalogram (EEG) data remains underexplored [1]. EEG-based brain-computer interfaces (BCIs) represent a critical domain within neurotechnology and rehabilitation. These systems enable non-invasive decoding of brain activity for communication or control purposes, particularly in individuals with neurological impairments [2–4]. Among the most widely adopted paradigms in EEG-based BCI is motor imagery (MI)–the mental rehearsal of movement without physical execution [5, 6]. MI-based BCIs allow users to control external devices simply by imagining limb movements, making them highly valuable in assistive technologies and neurorehabilitation contexts [7–9]. However, EEG signals collected during MI tasks are often subtle and complex, requiring advanced decoding techniques to extract task-relevant features with high accuracy. Additionally, EEG data present significant privacy concerns because they can reveal sensitive cognitive, emotional, and medical information, including aspects of user identity, and behavioral states [10]. If EEG data are not protected, developers or attackers may be able to extract sensitive information, including security details, such as passwords and ATM PINs [11]. Recent research in EEG based BCI has introduced several privacy-preserving techniques for example, encryption, federated learning, and perturbation. Among these approaches, encryption has been widely adopted because it provides effective privacy protection while preserving feature dimensionality through key-based transformation. As a result, recent studies have explored encryption techniques to protect EEG data in BCI systems [12–17].

From the literature review, it is clear that most existing privacy preserving encryption methods either reduce EEG signal fidelity, rely heavily on hand-crafted features, or require decryption during processing. This increases computational overhead and often reduces classification performance. Consequently, current approaches protect only isolated stages of the BCI workflow rather than providing seamless, end-to-end privacy-preserving representation learning suitable for real-time MI applications. To address these challenges, we propose a deep denoising structure-preserving neural network (DSNet) that enables model training and inference directly on privacy-preserving encoded data while maintaining the statistical geometry to distinguish between MI classes. The proposed DSNet framework addresses this gap by applying a key-modulated non-reversible neural encoding function that hides the raw feature values but preserves important relationships such as covariance and class separability. Because DSNet trains its neural classifier within the encoded feature space, the model learns to jointly denoise, extract discriminative patterns, and perform classification without reconstructing the original signal at any stage. As a result, DSNet provides effective privacy-preservation, maintains high classification accuracy, and is robust against adversarial inference, effectively addressing the key limitations of prior methods. While prior studies predominantly rely on formal cryptographic mechanisms or ciphertext-domain computation, the proposed framework takes a complementary representation-learning approach by generating non-reversible privacy-preserving encoded representations for direct downstream classification.

To further enhance clinical interpretability, the outputs of DSNet are integrated with a LLM to generate structured, clinical-style summaries. These summaries emulate neurologist-style diagnostic reports, translating numerical predictions into natural language interpretations. This integration bridges low-level neural decoding with high-level clinical reasoning, providing a privacy-preserving and interpretable AI pipeline for EEG-based BCI systems. The main contribution of this study can be summarized as follows:

- Introducing DSNet a novel framework that combines privacy-preserving neural encoding, learned denoising, and deep neural networks to enable accurate EEG decoding directly in the encoded feature space without requiring reconstruction of the original signal.
- Demonstrating superior performance of DSNet-NN in achieving high classification accuracy and low information loss across all evaluated subjects, outperforming existing benchmarks.
- Providing a computationally efficient and privacy-resilient solution capable of mitigating common attack vectors such as data breaches and interception threats.
- Integrating LLM-based clinical report generation, thereby enhancing the interpretability and usability of EEG classification outcomes in digital health and neurorehabilitation contexts.

The paper is structured as follows: “Proposed framework” Section provides a description of the proposed model framework. A detailed analysis of the results and a comparative discussion are provided in “Result” and “Discussion” Sections. Finally, this study concludes with the findings and future implications in “Conclusion” Section.

### Related works

This section provides an overview of recent studies applying encryption as a privacy-preserving strategy for EEG data. This literature review aligns directly with the objective of this study and helps to identify a well-defined research gap. Agarwal et al. [13] applied secure multiparty computation (SMC) to perform distributed linear regression across users without exposing raw EEG signals. Their results showed comparable RMSE to linear regression; however, the method relies on additive secret sharing and requires multiple communication rounds between users. It also supports only linear models, making it unsuitable for deep neural network-based EEG decoding. Similarly, Liu et al. [14] applied Paillier encryption to enable encrypted feed-forward neural network classification by replacing activation functions with polynomial approximations. While the method supports privacy-preserving encoded multi-class EEG classification, it introduced significant computational cost and led to a decrease in accuracy.

Likewise, Peng et al. [12] also explored Paillier-based homomorphic neural networks, enabling computation directly on ciphertext. However, the polynomial activation approximation leads to accuracy degradation, and computational cost increases quadratically with input dimensionality. In addition, these models typically support encrypted inference only; training still occurs on unencrypted data, exposing the model to privacy risks such as feature inversion and membership inference attacks.

Cancelable biometric systems such as PolyCosGraph [16] provide privacy by applying polynomial projection and cosine embedding to EEG connectivity graphs. This method demonstrated low EER across several conditions; however, it is intended for identity authentication, not continuous motor imagery decoding. It does not preserve class-discriminative neural patterns required for real-time BCI control, and it does not support encrypted model training.

More recently, Yan et al. proposed LightPyFE [15], a privacy preserving EEG feature extraction scheme under edge computing, where users retain encryption/decryption operations while secure floating-point and integer operations are offloaded to edge servers. Although this design reduces latency and prevents server-side exposure of raw EEG, LightPyFE performs feature extraction only, without supporting encrypted classification or end-to-end neural learning. It also assumes a semi-honest threat model, in which edge servers do not attempt to infer private information, which may not hold in applications requiring strong adversarial security guarantees. Because the privacy-preserving encoded data are not optimized for discriminability, classifier performance remains limited and model adaptation is not possible across different subjects or BCI sessions.

In addition, the AI-driven DD-FPE based framework [17] proposed a format-preserving encryption-based data desensitization (DD-FPE) mechanism integrated with CSP feature extraction and deep neural networks for MI classification. However, the encryption occurs before model training, meaning neural network optimization itself is not performed on privacy-preserving encoded data, preventing robust end-to-end representation learning.

The proposed DSNet framework, implemented as a feedforward neural network (NN) or a recurrent neural network (RNN), addresses these limitations by applying a non-reversible, structure-preserving neural encoding function that maintains the covariance and discriminative geometry of CSP-derived EEG features while obscuring raw feature values. Unlike prior methods, DSNet performs training directly within the encoded feature space, enabling joint denoising and MI classification without reconstructing the original signal at any stage. In this framework, denoising refers to the learned ability of the classifier to extract meaningful neural patterns from encoded input representations while suppressing distortions introduced by the encoding transformation. Consequently, DSNet enables end-to-end privacy-preserving MI classification while maintaining strong classification performance. Importantly, the proposed DSNet framework should be interpreted as a privacy-preserving neural encoding mechanism that generates non-reversible encoded representations for downstream learning, rather than as a formal cryptographic scheme with provable security guarantees. This representation-learning perspective complements existing encryption-based approaches while avoiding the computational burden typically associated with ciphertext-domain processing.

## Proposed framework

**Fig. 1:**
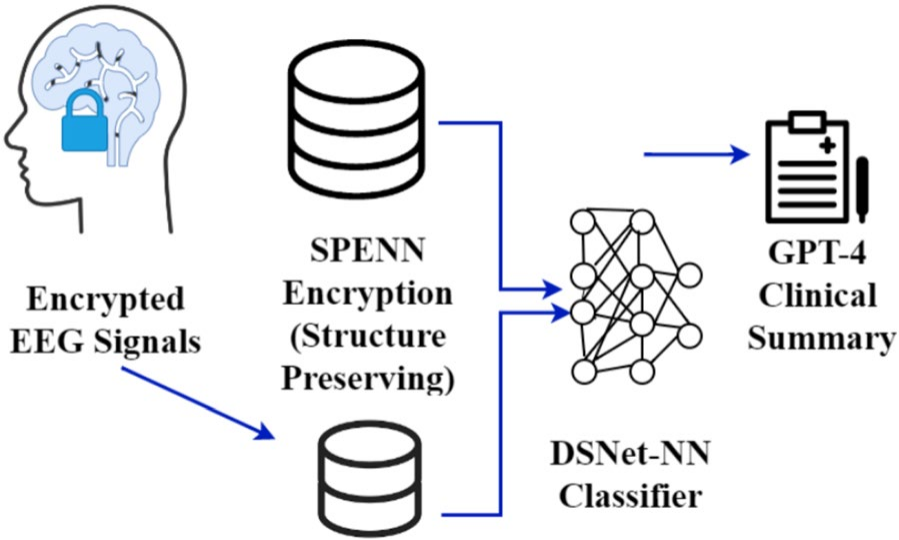
Overview of the proposed framework

We propose a deep learning pipeline that preserves privacy and is clinically interpretable for EEG-based BCI classification that incorporates both privacy-preserving encoded signal processing and LLM-based clinical reasoning. The architecture is designed to address key challenges in secure healthcare data analysis, enabling privacy-preserving EEG classification while generating natural-language outputs to support clinical decision-making. Figure 1 illustrates the proposed framework. This unified framework consists of six components: (1) dataset and experimental setup, (2) EEG preprocessing and feature extraction, (3) privacy-preserving encoded classification using DSNet, (4) LLM-based generation of clinical summaries, (5) performance evaluation using classification and interpretability metrics, and (6) assessment of resilience to privacy attacks.

### Dataset and experimental setup

**Fig. 2:**
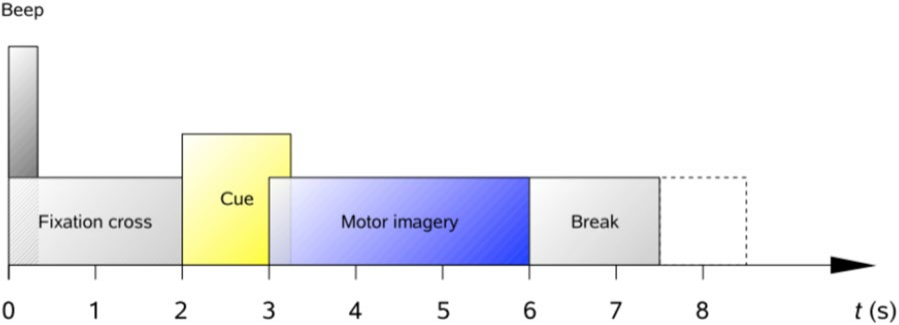
Timing scheme of the paradigm for MI dataset

We utilized a publicly available EEG dataset comprising recordings from nine subjects [18]. The dataset includes four MI tasks: imagination of left-hand movement (class 1), right-hand movement (class 2), foot movement (class 3), and tongue movement (class 4). During data collection, participants were seated comfortably in front of a screen. As illustrated in Fig. 2, each trial in the BCI Competition IV Dataset 2a or MI dataset follows a standardized 8-second sequence: a fixation cross and auditory beep (0–2 s), a visual cue indicating the MI task (2–3 s), a motor imagery period (3–6 s), and a rest phase (6–8 s). This consistent trial design facilitates the alignment and extraction of EEG features specific to motor imagery. Notably, no feedback was provided during the sessions. For this study, we focused on binary classification between left-hand and right-hand imagery (classes 1 and 2), aligning with practical BCI use cases in neurorehabilitation.

### EEG preprocessing and feature extraction

Raw EEG signals were bandpass filtered between 4 and 40 Hz using a fifth-order Butterworth filter [7]. Epochs were segmented from 0.5 to 3.5 s post-cue. Ocular artifacts were removed by discarding electrooculography (EOG) channels. Each subject’s EEG recording contained 72 trials per class 72 for left-hand MI (LH-MI) and 72 for right-hand MI (RH-MI) giving a total of 144 trials. During preprocessing, the EEG data were filtered into nine frequency bands ranging from 4 to 40 Hz with 4 Hz intervals (04–08, 08–12, …, 36–40 Hz). Each trial segment was extracted from 0.5 to 3.5 s after cue onset, covering a 3 s MI window. An 80/20 stratified random split was applied at the trial level to form the training and testing sets while preserving equal class proportions between left-hand and right-hand MI trials. This resulted in 58 trials per class (116 total) for training and 14 trials per class (28 total) for testing. Importantly, all data-driven preprocessing steps, including CSP spatial filter estimation, mutual information-based feature selection, and feature standardization, were performed using the training data only [7]. The learned transformations were then applied unchanged to the held-out test set to prevent information leakage and ensure reproducibility. From each band, the first and last two spatial filters (m = 2) were selected, producing 4 log-variance features per band. With nine bands, the concatenated feature vector for each trial contained 36 features in total (4 $\times$ 9). To identify the most discriminative features, mutual information (MI)-based feature selection (SelectKBest) was used, retaining all 36 dimensions since they were already the top-ranked informative features (based on average accuracy). Thus, the training and testing feature matrices had shapes of (116, 36) and (28, 36) respectively.

### DSNet framework for privacy-preserving classification

After MI-based feature selection, each MI trial produced a 36-dimensional feature vector across nine frequency bands (4–40 Hz in 4 Hz steps). For clarity, we denote each feature vector as:

1 $$\begin{aligned} \textbf{x} \in \mathbb {R}^{36} \end{aligned}$$

In Eq. (1), $\textbf{x}$ represents the feature vector for a single MI trial containing 36 log-variance values. For a representative subject, the dataset consisted of 144 trials (72 LH-MI and 72 RH-MI), which were split 80/20 into 116 training samples (58/58) and 28 testing samples (14/14). These feature vectors were then transformed into privacy-preserving encoded representations using the SPENN. The notion of structure preserving encoding originates in cryptographic research, where encoded representations are designed to maintain internal algebraic or relational structure required for computation while ensuring non-invertibility [19]. Inspired by this principle, the proposed SPENN module preserves key geometric properties of EEG feature space (e.g., mean-variance structure and covariance relationships) while preventing raw feature reconstruction. This enables privacy-preserving EEG classification without reconstructing the original signal. In this study, SPENN is not a conventional cryptographic cipher such as FPE; instead, it is a neural-network-based, non-reversible encoding model that generates privacy protected representations while preserving the statistical geometry necessary for downstream learning while protecting raw feature values. Specifically, SPENN applies a deterministic nonlinear transformation parameterized by a subject-specific key $K_s$:

2 $$\begin{aligned} S(\textbf{x}) = f_{\theta }(\textbf{x}, K_s) \end{aligned}$$

In Eq. (2), $S(\textbf{x})$ is the privacy-preserving encoded EEG feature representation and its output is used directly for training and classification, $f_{\theta }$ denotes a small feedforward neural network (Dense $\rightarrow$ ReLU $\rightarrow$ Dense), and $K_s$ modulates internal activations via key-based scaling and shifting. The subscript s in $K_s$ represents the subject-specific encoding key / parameter. The mapping is non-invertible, meaning the original feature vector $\textbf{x}$ cannot be reconstructed without knowledge of $K_s$. This produces an encoded representation of the same dimension:

3 $$\begin{aligned} S(\textbf{x}) \in \mathbb {R}^{36} \end{aligned}$$

Structure preservation is enforced by ensuring that the privacy-preserving encoded data retain similar mean and covariance properties to the original feature space:

4 $$\begin{aligned} \mathcal {L}_{struct} = \Vert \mu _{\textbf{x}} - \mu _{S(\textbf{x})} \Vert _{2} + \Vert \Sigma _{\textbf{x}} - \Sigma _{S(\textbf{x})} \Vert _{F} \end{aligned}$$

In Eq. (4), $\mathcal {L}_{\text {struct}}$ stands for structure-preserving Loss which ensures the encoded feature space retains the original statistical geometry (mean + covariance). $\mu _{\textbf{x}}$ is the statistical mean of the feature vector, $\mu _{S(\textbf{x})}$ is the mean of the encoded representations,$\Sigma _{\textbf{x}}$ is the Covariance matrix of original features, $\Sigma _{S(\textbf{x})}$ is the Covariance after encoding, $\Vert .\Vert _{F}$ is the Frobenius norm which measures difference between two covariance matrices. Privacy is enforced by penalizing reconstruction feasibility:

5 $$\begin{aligned} \mathcal {L}_{priv} = \text {ReconstructionPenalty}(\textbf{x}, S(\textbf{x})) \end{aligned}$$

In Eq. (5), $\mathcal {L}_{priv}$ stands for privacy loss which ensures encoding is non-invertible and prevents reconstruction of the original signal. The overall SPENN optimization objective is:

6 $$\begin{aligned} \mathcal {L}_{SPENN} = \mathcal {L}_{struct} + \alpha \mathcal {L}_{priv} \end{aligned}$$

In Eq. (6), $\mathcal {L}_{\text {SPENN}}$ stands for final training objective which balances structure preservation and privacy. Here, $\alpha$ is a weighting hyperparameter that controls the privacy-utility trade-off. In our implementation, $\alpha$ = 0.5 was selected empirically to preserve discriminative structure while ensuring non-invertibility of the encoded representations. Importantly, SPENN does not introduce random noise. The perturbations arise deterministically from key-based transformations, meaning that the encoded representations preserve class discriminative geometry but obscure exact numerical values. In DSNet, the encoding transformation introduces controlled perturbations (rather than random noise) to obscure original feature values. The role of DSNet is to learn to suppress these perturbations or encoding artifacts, preserve the underlying geometry and recover clean class-relevant structure. The encoded feature matrices (train: $116 \times 36$, test: $28 \times 36$) were standardized and passed directly to the DSNet classifiers without reconstructing the original signal.

These encoded features are then fed into classifiers (either a feedforward NN or a recurrent LSTM-based RNN). In this stage, the deep neural classifier performs joint denoising and classification, learning to suppress encoding-induced distortions while enhancing class-relevant MI patterns. The NN consisted of two Dense layers (64 units, ReLU), each followed by 50% dropout, and a final softmax output layer [20]. The RNN consisted of two stacked LSTM layers (64 units each, ReLU) with dropout between layers and a softmax output layer. Both models were trained for 50 epochs (batch size = 32) using the Adam optimizer and categorical cross-entropy loss. Because the models are trained entirely on encoded inputs, they learn to recover discriminative neural structure directly in the encoded feature space, enabling accurate MI classification without ever decrypting the data [21].

**Algorithm 1:**
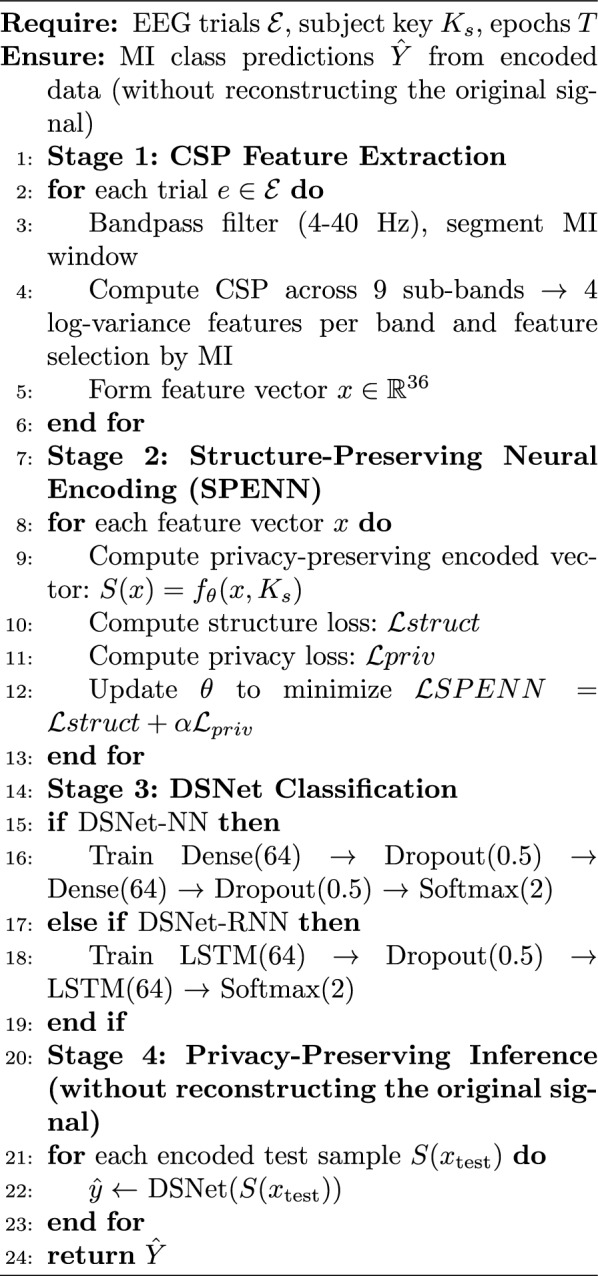
DSNet: deep denoising structure-preserving neural network

### LLM-based clinical output generation

To enhance interpretability and clinical usability, we integrated GPT-4 into the DSNet-NN/RNN pipeline to generate concise, clinical-style summaries of MI classification outcomes. Once the privacy-preserving encoded EEG data were classified by either the DSNet-NN or DSNet-RNN model, the predicted MI class, the corresponding softmax-derived average confidence scores, the predicted highest confidence score of the respective class and the model identifier were formatted into a structured prompt for GPT-4. This LLM-based interpretation layer mimics the tone and structure of neurologist reporting, translating numerical classification outputs into natural-language summaries suitable for clinical review.

After model inference, each test sample produced a probability vector:

7 $$\begin{aligned} \textbf{p} = [p_0, p_1], \qquad p_0 + p_1 = 1 \end{aligned}$$

where $p_0$ and $p_1$ denote the predicted probabilities for left-hand and right-hand MI, respectively. The predicted class was determined as:

8 $$\begin{aligned} \hat{y} = \arg \max (p_0, p_1) \end{aligned}$$

and the corresponding confidence score was computed as:

9 $$\begin{aligned} \text {Confidence}(\%) = 100 \times \max (p_0, p_1) \end{aligned}$$

Rather than averaging across all predictions which may dilute clinically meaningful patterns, we selected the highest-confidence sample per subject as the representative classification result. This reflects standard clinical reasoning practice, where the clearest neural response is considered more reliable for interpretation. Average class-wise confidence was retained only to provide contextual support. The computed outputs (predicted class, highest confidence score, average class confidence, and model accuracy) were formatted into a structured prompt and submitted to GPT-4 using a deterministic decoding strategy (temperature = 0) to ensure reproducibility. API keys were securely stored in environment variables. Two prompt components were used: (1) a system instruction defining clinical tone and (2) a user prompt populated with subject-specific classification results. We include below a complete, real prompt example used in the system, to ensure full transparency and reproducibility.

**Algorithm 2:**
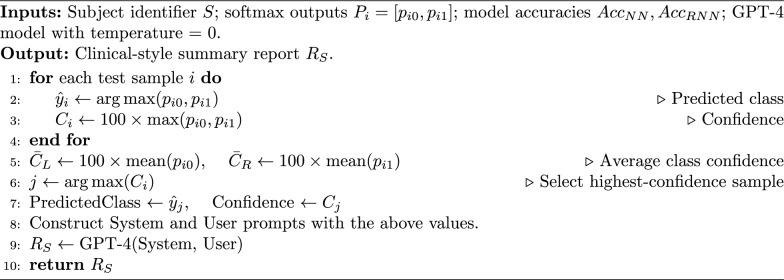
LLM-assisted clinical reporting

### Example prompt used for GPT-4 clinical summary generation

The following prompt template was used to generate clinical-style narrative summaries from the classification outputs of the DSNet-NN/RNN models. Model predictions and confidence scores were inserted into the template programmatically prior to querying GPT-4.
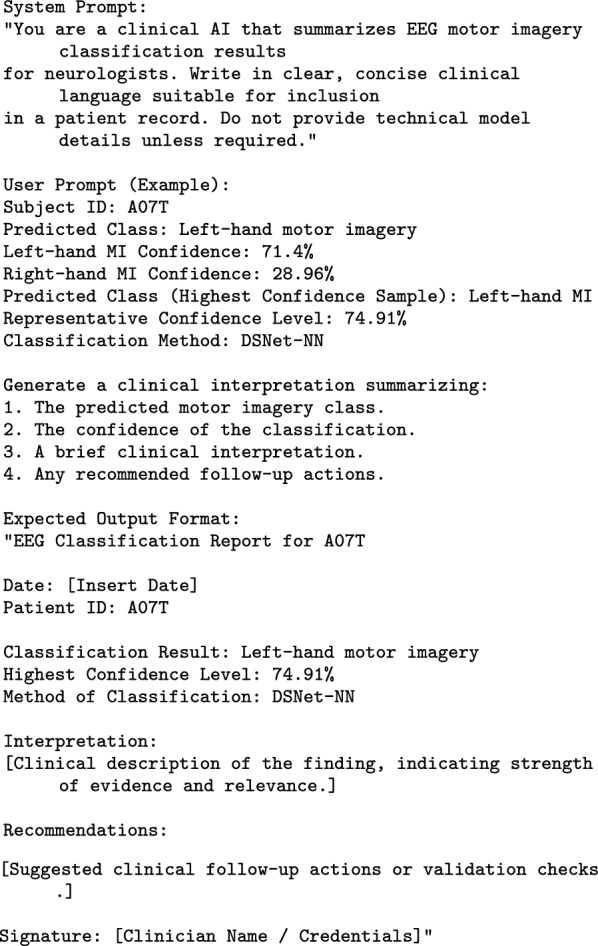


### Privacy threat resistance

The DSNet-NN/RNN framework protects EEG data using a non-invertible, structure preserving neural encoding process that maintains the statistical geometry required for classification while concealing physiologically meaningful signal content. Thus, even if encoded feature matrices are exposed, they cannot be interpreted, reconstructed, or used to infer cognitive state.

#### Threat model and attack simulation setup

To evaluate the privacy-preserving capability of the proposed framework, adversarial threat models were simulated under realistic attack assumptions. Importantly, all attack simulations were conducted independently for each subject, ensuring that privacy resistance is measured at the subject level rather than based on aggregated effects. We consider three practical adversarial scenarios commonly encountered in real-world BCI deployment:

- **Database Compromise (Data Breach):** An external attacker gains access to stored privacy-preserving encoded EEG feature representations.
- **Insider Threat (Semi-Honest Adversary):** A legitimate system user attempts to infer cognitive states or reconstruct original features, despite following the protocol.
- **Man-in-the-Middle (MITM) Interception:** Encoded feature representations are intercepted during transmission between client and server.

In all scenarios, the adversary has access only to encoded feature vectors. The attacker does not have access to raw EEG data, DSNet classifier weights, or subject specific encoding parameters. Thus, the attacker is constrained to operate strictly in the encoded feature space.

To quantitatively evaluate privacy resistance, two adversary models were implemented:

- **Label Inference (Surrogate Model) Attack:** Attempts to approximate the DSNet-NN decision boundary using privacy-preserving encoded data (with labels).
- **Structural Leakage (Feature Label Dependence) Attack:** Attempts to detect whether class structure remains embedded in the encoded feature space using only feature vectors (without labels).

### Evaluation metrics

Model performance and clinical interpretability and potential real-world applicability were assessed using: classification accuracy, root mean square error (RMSE), area under the ROC curve (AUC), computational time, and information loss. All performance metrics were computed **per subject** to evaluate the robustness and generalizability of both DSNet-NN and DSNet-RNN.

Attack success was evaluated based on:

- Classification accuracy relative to the 50% random baseline,
- ROC-AUC to assess class separability,
- Mutual Information (MI) ratio $k/d$ to quantify structural leakage, where $d = 36$ denotes the full feature dimension.

A successful privacy-preserving method is characterized by:

$$k/d \approx 0 \quad \text {and surrogate accuracy} \approx 50\%,$$

indicating negligible class-correlated leakage and resistance to reverse inference.

## Result

### Classification performance and information loss

To assess the effectiveness of the proposed framework under privacy-preserving constraints, we compared two architectures: a feedforward neural network (DSNet-NN) and a recurrent network (DSNet-RNN). Privacy-preserving encoded features were used as input for both models. Table 2 presents representative learning dynamics for the first five training epochs to illustrate convergence behavior and reduction in reconstruction-related loss during early optimization. In contrast, Table 1 reports the final subject-wise classification performance after completion of the full training procedure (50 epochs), and therefore reflects the final converged results used for comparative evaluation. Here, information loss refers specifically to the reconstruction-related RMSE, where lower values indicate better preservation of discriminative feature structure. As shown in Table 1, DSNet-NN consistently outperformed both the privacy-preserving encoded RNN and baselines (CSP-NN and CSP-RNN) across subjects A01T-A09T. DSNet-NN achieved classification accuracies ranging from 87.93 to 99.14%, with standard deviations indicating stable learning. It outperformed CSP-NN in seven of nine subjects and exceeded CSP-RNN in all cases, with relative gains ranging from 24.26 to 48.28%. In contrast, DSNet-RNN frequently underperformed, often failing to surpass 50% accuracy, highlighting its limited capacity to learn from privacy-preserving encoded EEG representations.

**Table 1:** Accuracy of the proposed DSNet compared to baseline methods for MI dataset

Subject	DSNet-NN		DSNet-RNN		CSP-NN		CSP-RNN	
	Acc (%)	SD	Acc (%)	SD	Acc (%)	SD	Acc (%)	SD
A01T	96.55	3.76	56.90	0.57	96.43	1.49	67.86	0.12
A02T	87.93	2.26	54.31	0.75	82.14	1.88	50.00	0.03
A03T	99.14	3.48	54.31	0.74	92.86	2.17	64.29	0.05
A04T	93.97	3.65	50.00	0.96	85.71	1.93	50.00	0.02
A05T	95.69	4.01	52.59	0.83	96.43	2.04	71.43	0.03
A06T	91.38	2.87	50.00	0.92	78.57	2.16	53.57	0.02
A07T	94.83	3.63	50.00	0.96	89.29	2.54	53.57	0.05
A08T	98.28	4.24	50.00	0.96	92.86	2.48	50.00	0.02
A09T	94.83	3.71	59.48	0.46	93.97	3.92	68.10	0.00

We further evaluated the learning dynamics across five training iterations. As illustrated in Table 2, DSNet-NN exhibited a consistent decline in information loss alongside increasing accuracy, demonstrating its capacity to extract meaningful representations from encoded inputs. In contrast, DSNet-RNN maintained a near constant information loss ( 0.69) across iterations with negligible accuracy improvement. These findings highlight the robustness and privacy-preserving capability of the DSNet-NN architecture.

**Table 2:** Information loss and accuracy across the first five iterations for DSNet-NN and DSNet-RNN

Subject	Approach	Iter 1		Iter 2		Iter 3		Iter 4		Iter 5	
		Loss	Acc (%)	Loss	Acc (%)	Loss	Acc (%)	Loss	Acc (%)	Loss	Acc (%)
A01T	DSNet-NN	0.71	53.44	0.75	62.06	0.56	73.27	0.50	75.00	0.46	77.58
	DSNet-RNN	0.69	55.17	0.69	51.72	0.69	49.13	0.68	53.44	0.68	47.41
A02T	DSNet-NN	0.85	54.31	0.90	47.41	0.76	56.03	0.72	56.03	0.76	48.27
	DSNet-RNN	0.69	44.82	0.69	46.55	0.69	39.65	0.69	55.17	0.69	43.96
A03T	DSNet-NN	0.98	53.44	0.75	60.34	0.61	63.79	0.55	74.13	0.52	72.41
	DSNet-RNN	0.69	57.75	0.69	42.24	0.69	41.37	0.69	40.51	0.69	42.24
A04T	DSNet-NN	0.87	52.58	0.61	68.10	0.58	66.37	0.60	62.93	0.56	69.82
	DSNet-RNN	0.69	49.13	0.69	50.00	0.69	50.86	0.69	53.44	0.69	51.72
A05T	DSNet-NN	0.78	50.86	0.73	49.13	0.68	62.06	0.65	57.75	0.58	72.41
	DSNet-RNN	0.69	44.82	0.69	41.37	0.69	49.13	0.69	43.10	0.69	50.86
A06T	DSNet-NN	0.78	51.72	0.75	55.17	0.71	57.75	0.63	62.06	0.54	70.68
	DSNet-RNN	0.69	43.10	0.69	51.72	0.69	56.03	0.69	60.34	0.69	51.72
A07T	DSNet-NN	0.67	64.65	0.59	68.96	0.61	68.96	0.50	75.00	0.46	78.44
	DSNet-RNN	0.69	47.41	0.69	42.24	0.69	52.58	0.69	53.44	0.69	52.58
A08T	DSNet-NN	0.79	49.13	0.68	58.62	0.57	73.27	0.51	70.68	0.46	74.13
	DSNet-RNN	0.69	50.86	0.69	49.13	0.69	47.41	0.69	50.00	0.69	54.31
A09T	DSNet-NN	0.74	59.48	0.72	56.89	0.59	69.82	0.57	70.68	0.52	75.86
	DSNet-RNN	0.69	49.13	0.69	56.03	0.69	55.17	0.69	55.17	0.69	51.72

**Fig. 3:**
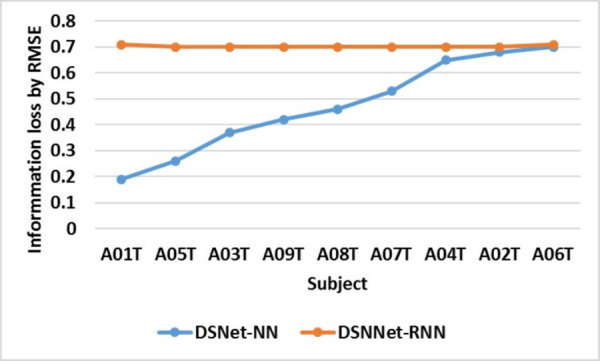
Information loss of DSNet-NN and DSNet-RNN algorithms based on average RMSE for MI dataset

Figure 3, illustrates the comparison of information loss, quantified using RMSE, across EEG subjects for two privacy-preserving EEG classification models: DSNet-NN and DSNet-RNN. The DSNet-RNN model exhibits a consistently high RMSE (approximately 0.70) across all subjects, indicating minimal ability to reduce information degradation introduced by encoding. In contrast, the DSNet-NN model demonstrates lower RMSE values for several subjects (e.g., A01T, A05T, A03T), suggesting improved retention of task-relevant features and effective denoising of encoded signals. Although RMSE increases for later subjects (e.g., A04T, A02T, A06T), DSNet-NN still maintains comparable or slightly better performance than DSNet-RNN.

**Fig. 4:**
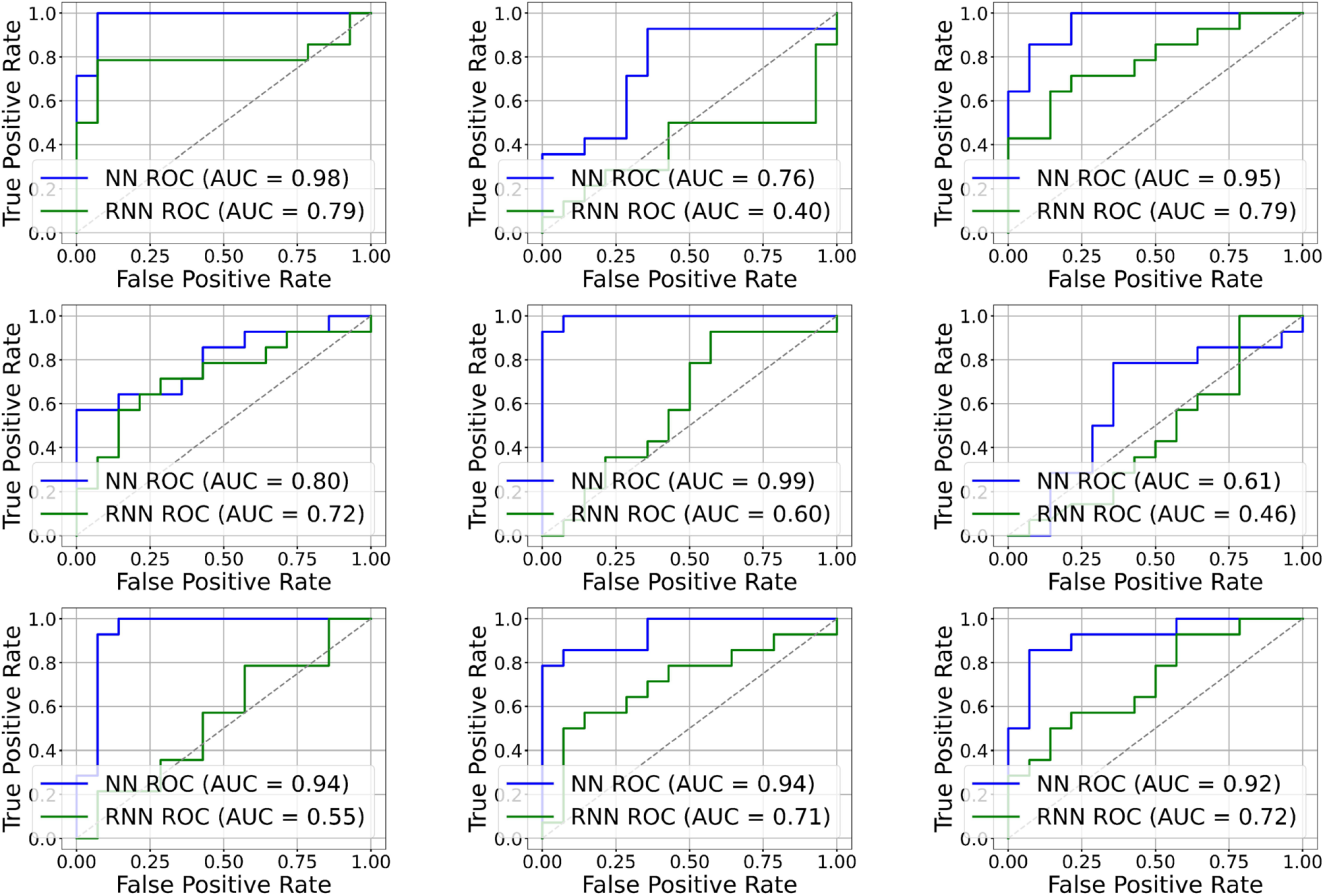
ROC curves of DSNet-NN and DSNet-RNN frameworks of all the subjects for MI dataset

In Fig. 4, The ROC curves across nine EEG subjects provide compelling evidence of the superior classification performance of DSNet-NN compared to its recurrent counterpart (DSNet-RNN). In each subplot, the ROC curve for DSNet-NN consistently outperformed those of the RNN model. Notably, the AUC values for DSNet-NN range from 0.61 to 0.99, with several subjects (e.g., A01T, A03T, A05T, A07T,and A08T) achieving near-perfect discrimination (AUC $\ge$ 0.94). In contrast, DSNet-RNN exhibits weaker performance, with AUCs clustering around 0.40 to 0.79.

### Computational efficiency

**Fig. 5:**
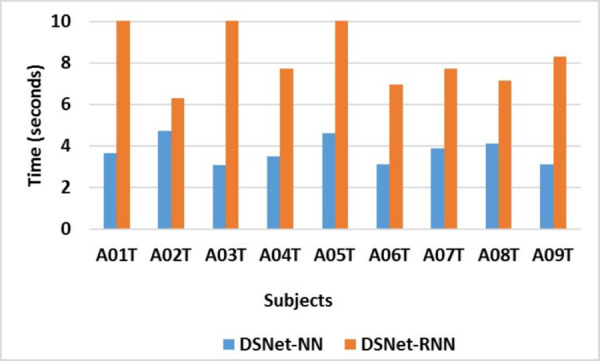
Performance of DSNet-NN and DSNet-RNN framework based on time for MI dataset

The bar chart in Fig. 5 illustrates the model execution time for each subject of MI dataset. DSNet-NN demonstrated significantly lower computational overhead, requiring only 3–5.5 s per subject, compared to 6.5–11.5 s for DSNet-RNN. The largest time difference was observed for subject A01T, where the RNN took more than three times longer than the NN. These results underscore the practical efficiency of DSNet-NN, for near real-time EEG classification in clinical settings.

**Table 3:** Accuracy comparison of privacy-preserving and non-privacy deep learning techniques for dataset MI2

Subject	DSNet-NN		DSNet-RNN		CSP-NN		CSP-RNN	
	Acc (%)	SD	Acc (%)	SD	Acc (%)	SD	Acc (%)	SD
S01	88.44	1.51	67.50	3.37	78.12	0.86	70.94	2.23
S02	89.53	1.45	62.81	3.07	72.50	3.40	66.88	2.24
S03	89.22	2.54	60.16	3.60	75.47	2.35	72.97	2.30
S04	88.44	0.77	55.00	2.13	74.69	2.19	69.69	0.91
S05	87.19	2.13	60.94	2.84	75.00	3.05	64.06	2.37
S06	90.94	0.80	61.25	2.35	68.75	4.42	61.25	1.53
S07	89.38	2.01	57.81	3.53	73.55	2.66	64.69	2.23
S08	89.53	1.06	58.75	4.06	71.09	2.47	58.91	1.45

**Fig. 6:**
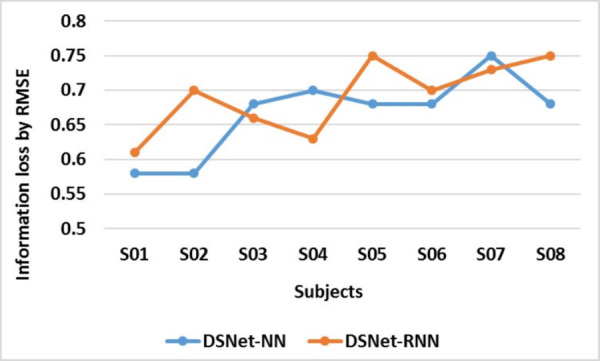
Information loss of DSNet-NN and DSNet-RNN algorithms based on average RMSE for MI2 dataset

In addition, we evaluated the proposed framework on the BNCI 2014-002 MI dataset to assess cross-dataset generalizability. This dataset includes two MI classes (left-hand vs right-hand), with 80 trials per class, collected using 15 EEG channels from 14 healthy subjects at 512 Hz. Detailed descriptions of the dataset are available in References [22]. We evaluated eight subjects from the MI2 dataset for classification accuracy, information loss (measured via RMSE), and clinical interpretability using LLM-generated summaries, following the same methodological procedure described in the proposed framework section. Table 3 presents the subject-wise accuracy comparison between DSNet-NN/RNN and baseline models for dataset MI2. The results show that DSNet-NN consistently achieved the highest classification accuracy across all subjects, outperforming DSNet-RNN and standard CSP-based classifiers. From Fig. 6, it is clear that DSNet-NN shows consistently lower information loss than DSNet-RNN across subjects, indicating stronger robustness to encoding effects. Both models maintain stable RMSE values, demonstrating reliable structure-preserving encoded EEG feature processing.

The performance comparison between the two privacy-preserving encoded classifiers indicates that DSNet-NN consistently outperforms DSNet-RNN across subjects for both the datasets. The lower performance of DSNet-RNN arises from the CSP-derived privacy-preserving encoded data, which are static spatial log-variance vectors and therefore do not contain temporal information. Since RNN/LSTM models depend on meaningful time-varying sequences to update memory states, the non-sequential input leads to underutilization of recurrent dynamics. Moreover, the structure-preserving encoding introduces deterministic key-based perturbations, which behave like pseudo-temporal noise and further disrupt LSTM state transitions. Together, these factors reduce training stability and result in comparatively weaker performance for DSNet-RNN. In contrast, DSNet-NN aligns naturally with the static encoded feature space and achieves higher classification stability.

### Clinical interpretability via LLM summaries

To support interpretable clinical decision-making, we integrated GPT-4 to generate concise, diagnostic-style reports from classification outputs. In this study, both quantitative and qualitative evaluations were conducted to comprehensively assess the reliability and clinical interpretability and potential applicability of the GPT-4 generated EEG summaries within the DSNet framework. As illustrated in Table 4, Subjects A01T-A05T demonstrated moderate RH-MI dominance, with highest confidence values ranging from 56 to 73%, indicating stronger right-hemispheric motor activation. In contrast, Subjects A06T-A09T showed highest LH-MI confidence (69 to 76%), reflecting stable left-hemispheric motor engagement. Subject A04T exhibited a balanced confidence profile between the two tasks. We have observed the same trend for MI2 dataset in Table 5. These confidence values guided GPT-4’s narrative synthesis, allowing the LLM to contextualize neural activation trends in clinically interpretable language. The generated summaries emulated neurologist-style phrasing, such as highlighting motor activation relevance and suggesting follow-up assessments when confidence was moderate.

In parallel, a quantitative analysis was performed using three widely recognized natural-language generation (NLG) metrics–BLEU, ROUGE-L, and BERTScore F1–to objectively assess the textual and semantic fidelity of GPT-4-generated clinical summaries [23–25]. Each generated report was compared against a structured reference template that captured clinically appropriate phrasing, terminology, and reporting conventions.

- BLEU (0-1): Measures n-gram overlap between generated and reference summaries, evaluating lexical precision. Higher BLEU values indicate closer surface-level phrasing alignment.
- ROUGE-L (0-1): Computes the longest common subsequence overlap, quantifying fluency and sentence-level recall of clinically relevant content. Higher values reflect stronger structural coherence.
- BERTScore F1 (0-1): Uses contextual embeddings from pre-trained transformers to capture semantic similarity between generated and reference texts. Higher values indicate better preservation of clinical meaning, even when wording differs.

**Table 4:** LLM-based clinical interpretation with confidence scores (%), predicted class (highest confidence score, (%)) and evaluation metrics for MI dataset

Subj	LH-MI	RH-MI	Pred. Class	GPT-4 clinical summary	ROUGE-L	BLEU	BERTScore F1
A01T	30.86	69.14	RH-MI 71.14	Moderate-confidence RH-MI activation; follow-up recommended for clinical validation and applicability	36.95	0.50	89.43
A02T	34.66	65.34	RH-MI 69.34	Suggests RH-MI neural activation; follow-up recommended for contextual validation	31.57	2.52	86.92
A03T	33.03	66.97	RH-MI 72.17	Strong RH-MI activation; clinical follow-up advised regarding rehabilitation relevance	31.48	2.01	87.24
A04T	47.59	52.41	RH-MI 56.14	Moderate-confidence RH-MI; additional evaluation recommended	35.05	2.25	87.75
A05T	27.44	72.56	RH-MI 73.55	RH-MI activation suggested; clinical evaluation advised	30.90	2.06	86.91
A06T	68.47	31.53	LH-MI 69.48	LH-MI activation detected; clinical correlation recommended	43.58	0.60	89.33
A07T	71.40	28.96	LH-MI 74.91	LH-MI activation evident; follow-up recommended	30.18	1.50	86.23
A08T	71.58	28.42	LH-MI 72.58	LH-MI pattern suggests intact motor planning, may support rehabilitation	34.95	2.10	87.25
A09T	75.90	24.10	LH-MI 76.92	LH-MI activity observed; further relevance assessment recommended	30.90	0.36	88.24

Across all subjects for both MI dataset (A01T-A09T) and MI2 dataset (S01-S08), the generated summaries achieved BLEU = 0.36−3.53, ROUGE-L = 30.18−43.58, and BERTScore F1 = 86.23−89.43 (Tables 4 and 5). These results demonstrate high semantic fidelity (BERTScore $F1 \ge 0.86$) and reasonable lexical and structural alignment (BLEU and ROUGE-L), confirming that GPT-4 effectively retained clinical meaning and interpretive intent despite stylistic variability inherent in NLG. By combining qualitative confidence-based interpretation and quantitative metric-based evaluation the DSNet-NN framework achieved both interpretability and linguistic rigor. Confidence driven inputs reflect neural certainty underlying each classification, while BLEU, ROUGE-L, and BERTScore objectively validate textual precision and semantic accuracy. Together, they confirm that DSNet-NN not only yields robust privacy-preserving encoded EEG classification but also produces LLM-based clinical summaries that are linguistically coherent, semantically faithful, and clinically meaningful.

**Table 5:** LLM-based clinical interpretation with confidence scores (%), predicted class (highest confidence score, (%)) and evaluation metrics for MI2 dataset

Subj	LH-MI	RH-MI	Pred class	GPT-4 clinical summary	ROUGE-L	BLEU	BERTScore F1
S01	46.40	53.60	RH-MI 56.34	EEG indicates RH-MI activation with moderate confidence; follow-up testing is recommended to improve reliability and explore additional motor or cognitive EEG tasks	43.07	3.50	88.38
S02	47.29	52.71	RH-MI 55.71	RH-MI neural activation detected; the relatively low confidence suggests further verification or clinical correlation may be required	43.08	3.53	88.35
S03	46.22	53.78	RH-MI 54.28	Strong RH-MI activation patterns observed; additional confirmatory assessments may enhance diagnostic confidence	43.08	3.53	88.35
S04	45.19	54.81	RH-MI 54.91	High-confidence RH-MI classification suggests robust neural engagement; further clinical interpretation may be beneficial	35.13	2.96	87.74
S05	55.74	44.26	LH-MI 56.25	Moderate LH-MI engagement observed; more data may be needed to support clinical interpretation	35.13	2.96	87.94
S06	62.81	37.19	LH-MI 63.92	LH-MI activity detected; clinical context required to determine relevance for motor function or rehabilitation planning	35.14	2.96	87.83
S07	39.61	60.39	RH-MI 63.40	RH-MI activation observed with moderate reliability; further diagnostic evaluation is advised	35.13	2.96	88.01
S08	56.31	43.69	LH-MI 59.41	LH-MI neural patterns present; additional monitoring may help confirm clinical significance	35.13	2.96	87.86

### Threat resistance

This section reports the results of the adversarial threat simulations described in the earlier section. As noted earlier, all attack simulations were performed independently for each subject, ensuring that the evaluation reflects subject-level privacy robustness rather than aggregated effects. Table 6 reports the range of attack models and evaluation outcomes across subjects for both the datasets. The findings demonstrate that DSNet-NN preserves the class-discriminative structure necessary for authorized classification while substantially reducing the exploitable information available to potential adversaries.

- **Label-Inference (Surrogate Model) Attack** **Attacker Knowledge:** privacy-preserving encoded data + labels (semi-honest insider). **Attack Target:** Approximate the DSNet-NN decision boundary without decrypting features. **Procedure:** For each subject, a surrogate neural classifier was trained directly on the encoded training set of size $116 \times 36$ and evaluated on encoded test data. The attacker has no access to model weights or the internal denoising representation learned by DSNet- NN. **Success Metric:** Classification accuracy and ROC-AUC. **Outcome:** Across subjects, surrogate models achieved ROC-AUC values in the range 0.50–0.60 and classification accuracy between 65.14 and 85.93%, which remained con- sistently lower than the authorized DSNet-NN classifier. This performance gap reflects the intended privacy-utility separation: DSNet-NN learns to suppress encoding-induced perturbations, whereas the attacker observes only encoded representations.
- ** Structural Leakage (Feature Label Dependence) Attack** **Attacker Knowledge:** encoded representations only (no labels). **Attack Target:** Determine whether class information remains embedded in the encoded feature space. **Procedure:** Mutual Information (MI) between privacy-preserving encoded data and labels was computed. The attacker selected the top $k=36$ MI-ranked dimensions and trained a weak surrogate classifier for each subject. **Success Metric:** MI ratio *k*/*d* (where $d = 36$ is the full feature dimension) and surrogate accuracy relative to the 50% random baseline. **Outcome:** The MI ratios were near zero ($k/d \approx 0$), and surrogate accuracy remained at 50% (chance level). This confirms that the developed encoding framework successfully removed class dependent structure, preventing feature inversion, reverse inference, or cognitive-state reconstruction.

**Table 6:** Attack models and evaluation outcomes

Attack type	Attacker knowledge	Objective	Metric	Outcome
Label inference (surrogate model)	Encoded representations + labels (semi-honest)	Recover class decision boundary	Accuracy, ROC-AUC	ROC-AUC $\ge 0.50 \le 0.60$; Accuracy $\ge 65.14 \le 85.93$%. Always lower than DSNet-NN
Structural leakage (MI-based)	Privacy-preserving encoded data only (no labels)	Detect residual class structure	MI ratio *k*/*d*, surrogate accuracy	$k/d \approx 0$; surrogate accuracy $\approx 50\%$ (chance). No class-correlated leakage

**Fig. 7:**
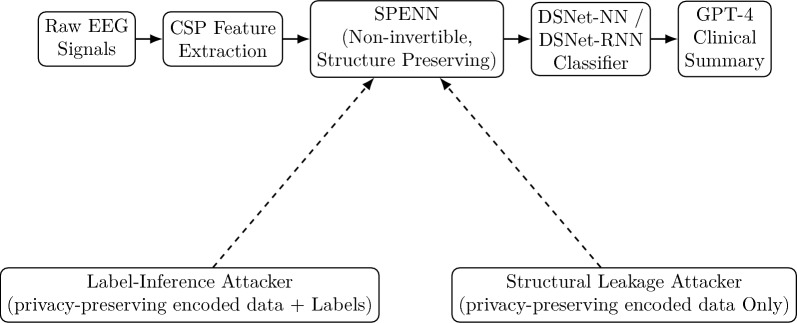
Threat model and attack flow. DSNet-NN preserves usability for classification while preventing adversaries from reconstructing class-relevant neural information

Figure 7 illustrates that DSNet-NN enables accurate privacy-preserving EEG classification for authorized users while preventing adversaries from reconstructing neural signals, identity, or cognitive state. The reduced surrogate performance and near-zero MI ratios confirm that DSNet-NN preserves statistical geometry necessary for learning while suppressing direct interpretability and inversion.

## Discussion

Although the LLM-generated summaries demonstrated strong semantic similarity to structured reporting templates, these findings should be interpreted as evidence of reporting-style consistency rather than direct clinical utility. The current evaluation supports interpretability and clinician-facing presentation of model outputs; however, prospective validation with practicing neurologists and real clinical workflows is required before establishing practical clinical usefulness. Beyond the interpretability component, this study presents a comparative analysis of two privacy-preserving deep learning models, DSNet-NN and DSNet-RNN for privacy-preserving EEG-based MI classification. Across all subjects (dataset MI and MI2), DSNet-NN consistently outperformed both its recurrent counterpart and non-privacy-preserving baselines. It achieved classification accuracies ranging from 87 to 99.14%, demonstrating robust generalizability across subjects and minimal degradation relative to baseline models. The feedforward model also exhibited better optimization dynamics, with progressive reductions in information loss and improved RMSE over training iterations, in contrast to the stagnant performance of DSNet-RNN. These findings establish the superiority of DSNet-NN for accurate and computationally efficient privacy-preserving EEG decoding, making it suitable for real-time healthcare applications.

Table 7 presents a subject-wise comparison of classification accuracy across five EEG-based MI classification methods evaluated on the BCI Competition IV 2a dataset. The proposed DSNet-NN model consistently outperforms existing privacy-preserving and federated learning approaches across all nine subjects. DSNet-NN achieves the highest accuracy for nearly all subjects, with performance ranging from 87.93 (A02T) to 99.14% (A03T). Compared to the previous state-of-the-art method DD-FPENN by Khanam et al.[17], which employed FPE, our developed DSNet-NN delivers marginal gains in most subjects, with improvements of up to 1.73% (A03T). This difference arises because DD-FPENN encrypts data before neural optimization, preventing the classifier learning directly from the encoded representations. In contrast, DSNet learns to denoise and classify entirely within the privacy-preserving encoded feature space, which strengthens discriminative performance.

Other models such as FedBS-EEGNet (Federated classification with local Batch-specific batch normalization and Sharpness-aware minimization) by Jia et al. [22] and MDMAML+SHOT+EEGNet (Multi-Domain Model-Agnostic Meta-Learning + source HypOthesis Transfer) by Li et al. [26] show significantly lower accuracies, particularly in subjects A02T and A05T, where performance drops below 40%. This suggests limitations in their ability to generalize across subjects. FedBS-EEGNet relies on cross-subject aggregation, which often suppresses subject-specific neural patterns and leads to reduced accuracy on individuals. DSNet maintains per-subject adaptation, preserving individualized MI characteristics. Similarly, MDMAML+SHOT+EEGNet assumes that spatial EEG patterns transfer well between subjects, which is often not the case due to strong inter-subject variability. DSNet instead learns directly from encoded representations unique to each individual. The PAW+EEGNet method by Huang et al. shows moderate results but lacks full coverage across subjects [27].

**Table 7:** Comparison of subject-wise accuracy between DSNet-NN and existing privacy-preserving EEG classification methods

Author	Model	A01T	A02T	A03T	A04T	A05T	A06T	A07T	A08T	A09T
Proposed model	DSNet-NN	**96**.**55**	**87**.**93**	**99**.**14**	**93**.**97**	**95**.**69**	**91**.**38**	**94**.**83**	**98**.**28**	**94**.**83**
Khanam et al. (2024)	DD-FPENN	95.69	93.10	97.41	93.10	94.83	92.24	98.28	96.55	93.97
Jia et al. (2024)	FedBS-EEGNet	68.52	28.96	74.31	44.39	36.69	42.77	49.28	68.11	66.75
Li et al. (2022)	MDMAML+SHOT+EEGNet	62.22	29.68	75.21	45.81	29.52	40.30	43.92	71.67	69.80
Huang et al. (2024)	PAW + EEGNet	66.10	41.20	78.80	62.90	59.10	56.30	–	–	62.20

The highest accuracy values are shown in bold

Overall, DSNet-NN demonstrates superior and stable classification accuracy, validating its effectiveness as a privacy-preserving and structurally robust solution for MI classification tasks in BCI applications. A key innovation of this study is the integration of LLMs for clinical interpretability. By embedding GPT-4 into the pipeline, we translated classification outcomes into concise, diagnostic style summaries that approximate the narrative style of neurologist reports. These LLM-generated texts enhance transparency, enable human-in-the-loop validation, and help bridge the gap between algorithmic decision-making and clinical workflows–addressing one of the core themes of this journal’s special issue. The proposed framework thus aligns with the broader vision of responsible and interpretable AI in healthcare, combining privacy preservation, model utility, and natural-language clinical reasoning. By supporting privacy-preserving EEG classification and interpretable summarization without compromising accuracy, DSNet provides a scalable and ethically grounded solution for integrating biosignal analysis with LLM–driven clinician-facing reporting systems.

## Conclusion

This study proposes DSNet, a novel deep learning framework for privacy-preserving and interpretable EEG classification using non-reversible encoded representations in BCI applications. By combining CSP-based feature extraction with structure-preserving encoding and deep neural network architectures, DSNet particularly its feedforward variant (DSNet-NN) demonstrated the ability to accurately decode EEG signals without the need for decryption. This approach effectively reconciles the trade-off between maintaining data privacy and ensuring high model utility. Experimental results across seventeen subjects from publicly available MI datasets revealed that DSNet-NN consistently outperformed both its recurrent counterpart and baseline models, achieving high classification accuracy, reduced information loss, and robust resistance to simulated privacy attacks. These findings support its potential for deployment in real-time neurorehabilitation settings and other healthcare applications involving sensitive neural data.

A key contribution of this framework lies in its integration with a LLM, GPT-4, which enables the generation of clinically interpretable summaries. This LLM-enhanced layer facilitates natural-language reporting of classification outcomes, aligning model outputs with the expectations of clinical decision support systems and improving the transparency of AI-based neurotechnology. Future research will expand this framework to incorporate additional EEG and multimodal neural signal representations, explore transformer-based and hybrid deep architectures, and integrate privacy-preserving federated learning to support distributed clinical deployment. Furthermore, while the present study provides rigorous quantitative and qualitative evaluation of the LLM-generated summaries using established NLG metrics, a blinded clinician assessment study is planned to validate the clinical interpretability, reliability, and real-world usability of the generated reports in practical healthcare and neurorehabilitation settings.

## Data Availability

The datasets used in this study are publicly available. It can be accessed at: https://www.bbci.de/competition/iv/#dataset2a and https://bnci-horizon-2020.eu/database/data-sets
